# The Small-Compound Inhibitor K22 Displays Broad Antiviral Activity against Different Members of the Family Flaviviridae and Offers Potential as a Panviral Inhibitor

**DOI:** 10.1128/AAC.01206-18

**Published:** 2018-10-24

**Authors:** Obdulio García-Nicolás, Philip V'kovski, Nathalie J. Vielle, Nadine Ebert, Roland Züst, Jasmine Portmann, Hanspeter Stalder, Véronique Gaschen, Gabrielle Vieyres, Michael Stoffel, Matthias Schweizer, Artur Summerfield, Olivier Engler, Thomas Pietschmann, Daniel Todt, Marco P. Alves, Volker Thiel, Stephanie Pfaender

**Affiliations:** a Institute of Virology and Immunology, Bern and Mittelhäusern, Switzerland; b Department of Infectious Diseases and Pathobiology, Vetsuisse Faculty, University of Bern, Bern, Switzerland; c Graduate School for Cellular and Biomedical Sciences, University of Bern, Bern, Switzerland; d Spiez Laboratory, Spiez, Switzerland; e Division of Veterinary Anatomy, Department of Clinical Research, Vetsuisse Faculty, University of Bern, Bern, Switzerland; f Institute for Experimental Virology, Twincore Centre for Experimental and Clinical Infection Research, Hannover, Germany; g Department of Molecular and Medical Virology, Ruhr-University Bochum, Bochum, Germany

**Keywords:** flavivirus, pestivirus, hepacivirus, K22, antiviral, panviral inhibitor, Flaviviridae

## Abstract

The virus family Flaviviridae encompasses several viruses, including (re)emerging viruses which cause widespread morbidity and mortality throughout the world. Members of this virus family are positive-strand RNA viruses and replicate their genome in close association with reorganized intracellular host cell membrane compartments. This evolutionarily conserved strategy facilitates efficient viral genome replication and contributes to evasion from host cell cytosolic defense mechanisms. We have previously described the identification of a small-compound inhibitor, K22, which exerts a potent antiviral activity against a broad range of coronaviruses by targeting membrane-bound viral RNA replication. To analyze the antiviral spectrum of this inhibitor, we assessed the inhibitory potential of K22 against several members of the Flaviviridae family, including the reemerging Zika virus (ZIKV). We show that ZIKV is strongly affected by K22. Time-of-addition experiments revealed that K22 acts during a postentry phase of the ZIKV life cycle, and combination regimens of K22 together with ribavirin (RBV) or interferon alpha (IFN-α) further increased the extent of viral inhibition. Ultrastructural electron microscopy studies revealed severe alterations of ZIKV-induced intracellular replication compartments upon infection of K22-treated cells. Importantly, the antiviral activity of K22 was demonstrated against several other members of the Flaviviridae family. It is tempting to speculate that K22 exerts its broad antiviral activity against several positive-strand RNA viruses via a similar mechanism and thereby represents an attractive candidate for development as a panviral inhibitor.

## Abstract (precis)

The virus family Flaviviridae encompasses several viruses, including (re)emerging viruses which cause widespread morbidity and mortality throughout the world. Members of this virus family are positive-strand RNA viruses and replicate their genome in close association with reorganized intracellular host cell membrane compartments.

## INTRODUCTION

Members of the family Flaviviridae are of major human health concern, and in several cases, the development of effective options for the prevention and treatment of infections caused by these viruses is urgently awaited. The Flaviviridae family comprises a wide variety of enveloped viruses possessing an RNA genome of positive polarity, and it is subdivided into four genera: Hepacivirus, Flavivirus, Pegivirus, and Pestivirus (1). While several new hepaciviruses were recently discovered in a variety of species (2), the most prominent member, hepatitis C virus (HCV), is responsible for chronic infection in >71 million individuals worldwide, which are at risk of developing liver cirrhosis and hepatocellular carcinoma (3). The genus Flavivirus encompasses 53 species and comprises a number of vector-borne, zoonotic agents responsible for acute and self-limiting diseases, which can, in some cases, lead to severe symptoms (vascular leakage, hemorrhage, encephalitis, meningitis) (4). As has been observed for Zika virus (ZIKV), which has recently spread throughout South and Central America and the Caribbean (5), several flaviviruses are considered emerging or reemerging pathogens and can rapidly become endemic, thereby leading to serious short- or long-term health consequences (6). Hepaciviruses and flaviviruses are therefore a major human health concern. The newly proposed genus Pegivirus as well as the genus Pestivirus mainly comprises virus species of veterinary relevance that cause gastrointestinal, respiratory, and reproductive diseases in animals, which are associated with major economic losses (7–9).

With the exception of HCV, intervention strategies against members of the family Flaviviridae are limited. This emphasizes the need for the development of effective and reliable drugs and vaccines, especially in the context of newly emerging or reemerging viral infections.

Members of the Flaviviridae family share a highly similar genome organization and replication strategy. Following attachment to the surface of the host cell and binding of the cellular entry receptor, viral particles are internalized via the endocytic route and incoming genomes are released into the cytosol. A single open reading frame (ORF) encodes both structural proteins composing the viral particles (capsid/core, prM, and envelope glycoproteins) (10) and nonstructural proteins primarily forming the viral replicase complex that ensures polyprotein processing, membrane reorganization, and RNA synthesis functions. Upon translation, the viral replicase is inserted in endoplasmic reticulum membranes, where it orchestrates the establishment of replication organelles. These organelles provide privileged, membrane-protected sites with which viral RNA synthesis is closely associated (11). Given that virus-induced membrane remodeling is a conserved mechanism among virtually all positive-sense RNA viruses, it represents an attractive target for the development of panviral inhibitors effective against a wide range of viruses.

Recently, a screening which aimed at identifying anti-human coronavirus 229E (HCoV-229E) compounds resulted in the identification of a potent inhibitor, K22, which efficiently abolished HCoV-229E plaque formation (12). The compound has been shown to act on early postentry stages of coronavirus replication and to target membrane-bound viral RNA synthesis. Moreover, K22 strongly prevented the formation of typical HCoV-229E-induced perinuclear double membrane vesicle (DMV) clusters. A study by Lundin et al. further extended the initial finding of the mechanism by which K22 inhibits HCoV-229E replication and demonstrated a potent pancoronavirus antiviral activity of K22 (12). Indeed, K22 inhibited a broad range of coronaviruses from several phylogenetic lineages (alpha-, beta-, and gammacoronaviruses), including murine hepatitis virus (MHV), type I feline coronavirus (FCoV), and avian infectious bronchitis virus (IBV), as well as the highly pathogenic human severe acute respiratory syndrome-associated coronavirus (SARS-CoV) and Middle East respiratory syndrome coronavirus (MERS-CoV) (12). Furthermore, it was recently reported that K22 impairs the replication of not only viruses in the Coronavirinae subfamily but also members of the Torovirinae subfamily, such as white beam virus (WBV; genus Bafinivirus) and equine torovirus (EToV; genus Torovirus), as well as porcine reproductive and respiratory syndrome virus (PRRSV) and equine arteritis virus (EAV), which are representative arteriviruses (13).

Here we evaluated the potential of K22 to inhibit the replication of members of the family Flaviviridae. We show that K22 efficiently impairs the replication of ZIKV, yellow fever virus (YFV), Japanese encephalitis virus (JEV), and West Nile virus (WNV), as well as, to a certain extent, Usutu virus (USUV), Wesselsbron virus (WESSV), HCV, and bovine viral diarrhea virus (BVDV). Collectively, our study indicates a broad antiviral activity of K22 against a wide range of Nidoviridae and Flaviviridae lineages and suggests that interfering with conserved mechanisms of membrane rearrangements and the biogenesis of replication organelles could represent a novel target for the development of antiviral drugs to combat positive-sense RNA virus infections.

## RESULTS

### K22 inhibits ZIKV in a dose-dependent manner.

**FIG 1:**
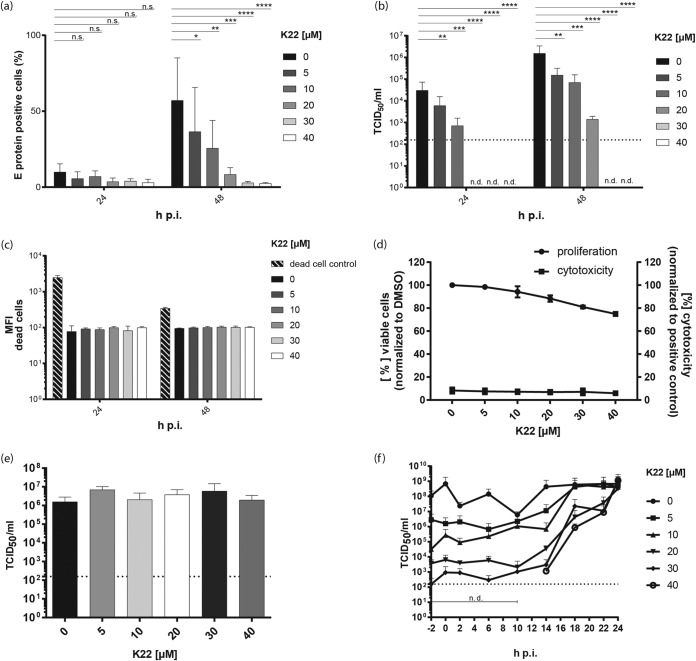
K22 inhibits ZIKV replication and acts at a postentry stage. Vero cells were treated for 4 h with different concentrations of K22. Cells were infected with ZIKV (MOI = 0.1 TCID_50_/cell) for 1 h before the medium was removed and medium containing the different concentrations of K22 was added. (a) At 24 h and 48 h p.i., cells were harvested and E protein expression was analyzed by flow cytometry. (b) Viral infectivity was determined by harvesting the supernatant of cells and performing a TCID_50_ assay on Vero cells. (c) Cells treated with the compound were stained with a live/dead cell marker, and the MFI was determined by flow cytometric analyses. Shaded bars represent the positive control of dead cells. (d) Commercially available cell toxicity assays (cytotoxicity; CytoTox 96 nonradioactive cytotoxicity assay; Promega) as well as viability assays (proliferation; MultiTox-Fluor multiplex cytotoxicity assay; Promega) were performed to determine the cytotoxic effect of K22 on Vero B4 cells at 24 h posttreatment. (e) To evaluate the direct effect of K22 on viral stability, ZIKV in DMEM was incubated for 2 h at room temperature with the different K22 concentrations, and the viral titer was determined as the number of TCID_50_ per milliliter. (f) Time-of-addition assays were performed to analyze the effect of K22 on the early virus-cell interaction. K22 at various concentrations was added at specific time points relative to the time of infection with ZIKV, and the cell culture supernatants were collected at 24 h p.i. for measurement of virus release. The results represent the mean (bar) + SD (*n* = 2 or 3). Dotted lines indicate the limit of detection. n.d., not detected. *P* values were determined by 2-way ANOVA, followed by Dunnett's multiple-comparison test. ****, *P* ≤ 0.0001; ***, *P* ≤ 0.001; **, *P* ≤ 0.01; *, *P* ≤ 0.05; n.s., not significant.

Given our previous findings that the small-compound inhibitor K22 may interfere with conserved features of virus-induced membrane remodeling, we hypothesized that K22 might, in addition to coronaviruses and arteriviruses, impair the replication of viruses belonging to the family of Flaviviridae, such as ZIKV. To test this hypothesis, K22-treated cells were infected with ZIKV and virus replication was assessed at 24 and 48 h postinfection (p.i.) by measuring the intracellular levels of E proteins by flow cytometry. The percentage of cells harboring replicating virus, reflected by E protein expression, was significantly decreased, in a dose-dependent manner, upon treatment of cells with K22 at 48 h p.i. (Fig. 1a). Titration of the viral supernatant confirmed the significant inhibition of ZIKV infectivity upon K22 treatment at both time points, i.e., at 24 and 48 h p.i., with 50% inhibitory concentrations (IC_50_s) of 2.5 μM and 2.1 μM, respectively (Table 1). Indeed, 5 or 10 μM K22 was sufficient to reduce the viral titers by 1 to 2 orders of magnitude. Higher concentrations of K22 reduced viral titers by up to 4 orders of magnitude (Fig. 1b). Flow cytometric analysis revealed no cytotoxic effect of K22 at the concentrations indicated in Fig. 1c, as determined upon application of a Fixable Aqua dead cell stain kit. These results were confirmed upon assessment of cytotoxicity using a commercially available kit (Fig. 1d). However, similar to what has been previously reported (12), K22 concentrations of ≥30 μM mildly affected cell proliferation (Fig. 1d; see also Fig. S1 in the supplemental material), suggesting that the observed antiviral effects at concentrations of ≥30 μM were slightly overestimated. In order to determine which step of the viral replication cycle is affected by K22, we performed time-of-addition experiments. To exclude the possibility of direct effects of K22 on viral particles, e.g., disruption of the viral envelope or interference with virion stability, we preincubated ZIKV with increasing K22 concentrations and subsequently assessed the remaining infectivity by titration. We showed that preincubation of K22 does not affect ZIKV infectivity (Fig. 1e). Next, K22 was added to the cells at different times pre- or postinfection, and the effects on viral replication and infectivity were determined by titration of the viral supernatant. Interestingly, K22 also displayed antiviral activity when added at several hours postinfection, suggesting an effect on postentry stages of the ZIKV life cycle (Fig. 1f). Collectively, our data reveal that K22 significantly inhibits ZIKV in a dose-dependent manner, likely by impairing a critical postentry step in the ZIKV life cycle.

**TABLE 1:** IC_50_ values and selectivity indexes for the effect of K22 treatment on flavivirus infectivity

Virus	24 h p.i.		48 h p.i.	
	IC_50_ (μM)	SI^*a*^	IC_50_ (μM)	SI
ZIKV	2.5	35.6	2.1	39.9
JEV	0.5	193.9	4.4	19.1
YFV	3.7	24.3	2.3	36.7
WNV	2.8	32.3	8.4	10.0
USUV	9.0	10.0	∼10.0	∼8.4
WESSV	5.9	15.2	6.8	12.5

a SI, selectivity index.

### K22 inhibits several members of the family Flaviviridae.

**FIG 2:**
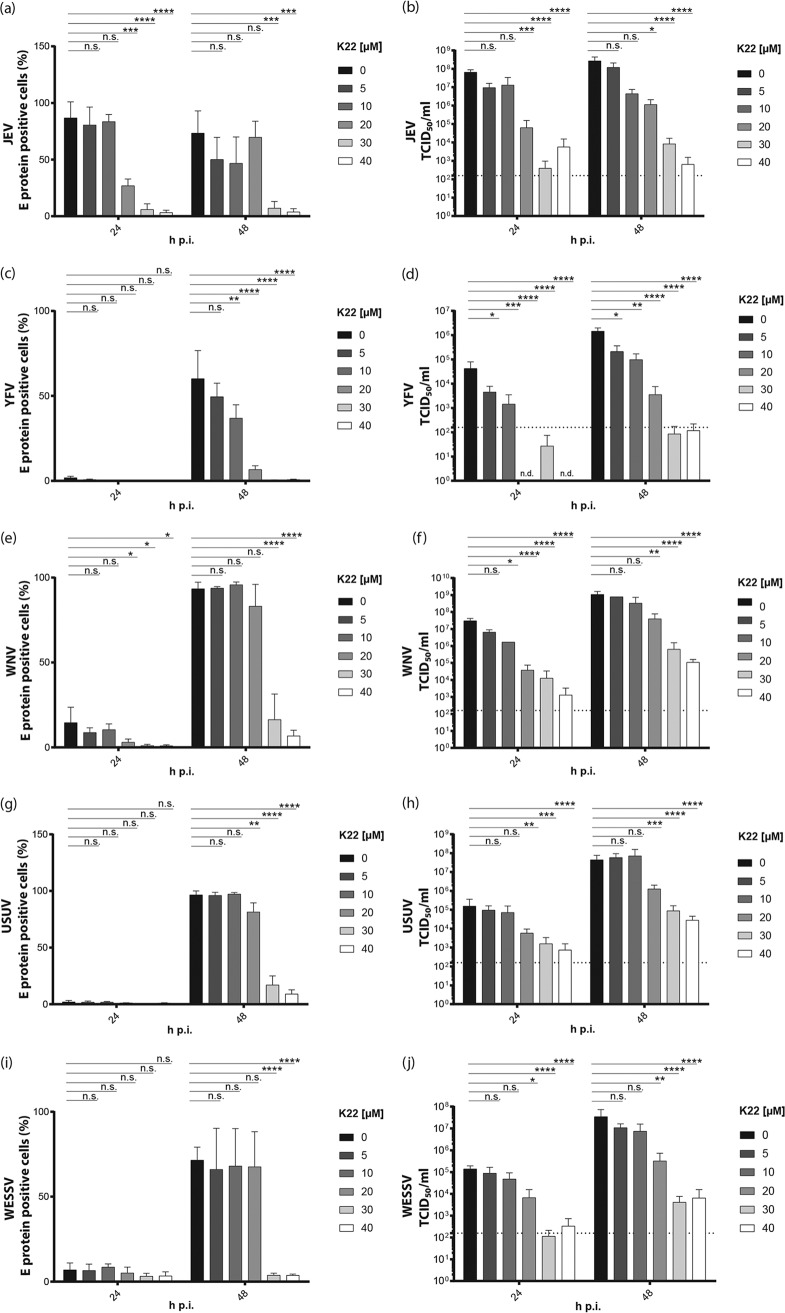
K22 inhibits the replication and infectivity of several members of the Flavivirus genus. Vero cells were treated for 4 h with different concentrations of K22. Cells were infected with JEV (a, b), YFV (c, d), WNV (e, f), USUV (g, h), and WESSV (i, j) (MOI = 0.1 TCID_50_/cell) for 1 h before the medium was removed and fresh medium containing the different concentrations of K22 was added. At 24 h and 48 h postinfection, cells and supernatants were harvested. (a, c, e, g) Cells were stained for anti-flavivirus group antigen antibody, and the percentage of positive cells was determined by flow cytometric analysis; (b, d, f, h) viral infectivity was determined from the supernatants of infected cells by performing a TCID_50_ assay on Vero cells. The results represent the mean (bar) + SD (*n* = 3). The dashed lines indicate the limit of detection. *P* values were determined by 2-way ANOVA followed by Dunnett's multiple-comparison test. ****, *P* ≤ 0.0001; ***, *P* ≤ 0.001; **, *P* ≤ 0.01; *, *P* ≤ 0.05; n.s., not significant.

**FIG 3:**
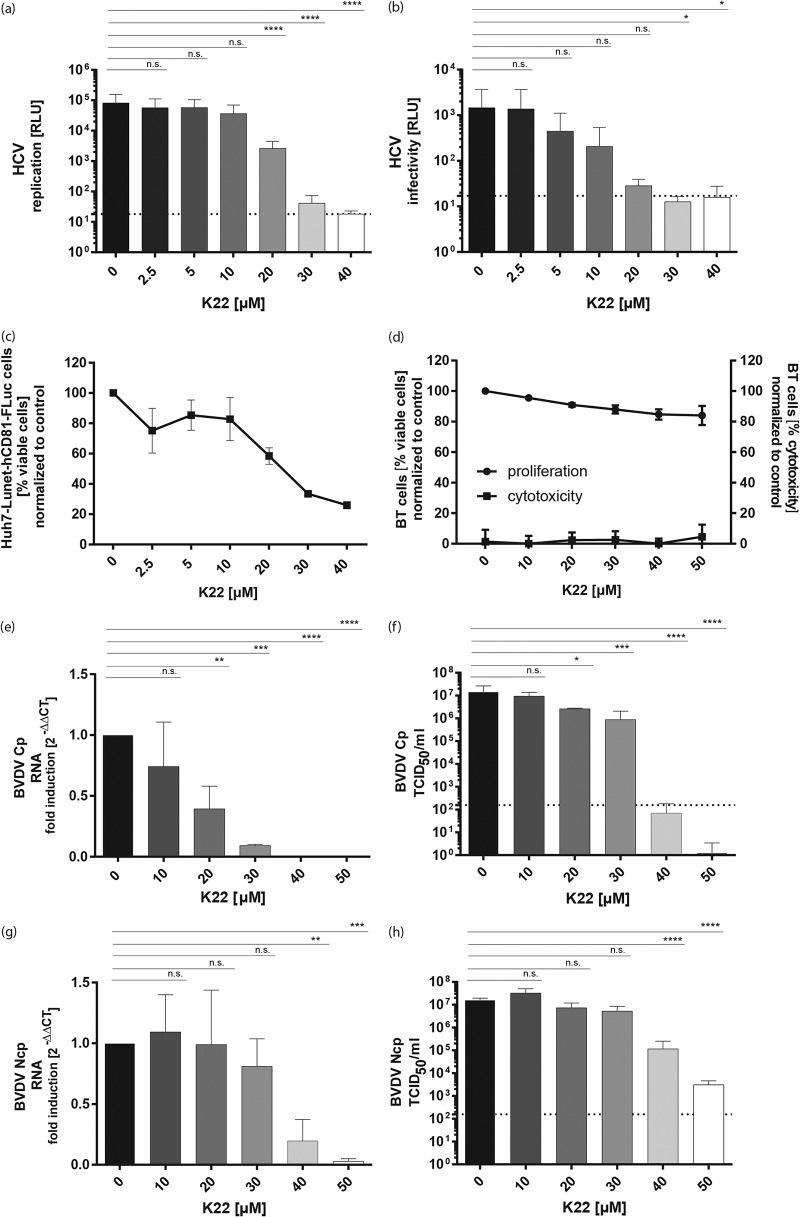
K22 inhibits the replication and infectivity of hepacivirus and pestivirus. Huh7-Lunet-N-hCD81-FLuc cells were pretreated with K22 for 3 h before supernatant removal and infection with JcR-2a virus for 3 h at 37°C. After this, the inoculum was removed and the compounds were added and left until the end of the experiment. (a) At 48 h p.i., the cells were lysed and Renilla luciferase (replication) activity was measured. (b) Infectious supernatants were harvested and transferred onto target Huh7-Lunet-N-hCD81-FLuc cells. Target cells were lysed at 72 h p.i., and Renilla luciferase activity (infectivity) was determined. Data are depicted as relative light units (RLU). (c) The viability of Huh7-Lunet-N-hCD81-FLuc cells after K22 treatment was determined upon MTT assay, and the data were normalized to those for untreated cells. Depicted are the data normalized to those for untreated cells. (d) The viability of BT cells after K22 treatment was determined using commercially available cell toxicity assays (cytotoxicity; CytoTox 96 nonradioactive cytotoxicity assay; Promega) as well as viability assays (proliferation; MultiTox-Fluor multiplex cytotoxicity assay; Promega). Depicted are the data normalized to those for the DMSO-treated cells. (e to h) BT cells were treated with K22 for 4 h before supernatant removal and infection with BVDV SuwaCp (e, f) or BVDV SuwaNcp (g, h) for 1 h at 37°C before the medium was removed and medium containing the different concentrations of K22 was added. (e, g) Cells were lysed at 48 h p.i., and intracellular RNA was extracted. qRT-PCR was performed, and viral replication was calculated using the ΔΔ*C_T_* method and is depicted as the fold induction compared to that for DMSO-treated control cells. (f, h) Viral titers were determined by serial dilution on BT cells at 4 days p.i. The results represent the mean (bar) + SD (*n* = 3). The dashed lines indicate the limit of detection. *P* values were determined by 1-way ANOVA followed by Dunnett's multiple-comparison test. ****, *P* ≤ 0.0001; ***, *P* ≤ 0.001; **, *P* ≤ 0.01; *, *P* ≤ 0.05; n.s., not significant.

We next assessed whether K22 displays a broad antiviral effect on the replication of other Flaviviridae members. To this end, we tested the K22 sensitivity of different flaviviruses, including JEV, YFV, WNV, USUV, and WESSV, as well as the hepacivirus HCV and the two representative pestivirus BVDV biotypes (cytopathogenic [Cp] and noncytopathogenic [Ncp]) of a bovine viral diarrhea pestivirus pair. Cells were treated with increasing concentrations of K22 and infected. Intracellular levels of the flavivirus E protein, as a marker of viral replication, were assessed by flow cytometry. Moreover, viral titers in the supernatant were determined by 50% tissue culture infective dose (TCID_50_) titration. This showed that both JEV and YFV replication (Fig. 2a and c) and infectivity (Fig. 2b and d) were reduced by K22 concentrations starting from 5 μM K22 in a dose-dependent manner, with IC_50_s being between 0.5 and 4.4 μM (Table 1). WNV (Fig. 2e and f) replication as well as infectivity was significantly reduced at K22 concentrations of ≥10 μM, with IC_50_s being 2.8 μM at 24 h and 8.4 μM at 48 h (Table 1). Slightly higher K22 concentrations (≥20 μM) were necessary to significantly inhibit USUV (Fig. 2g and h) and WESSV (Fig. 2i and j), with IC_50_s being between 5.9 and 10 μM (Table 1). To analyze the antiviral effect of K22 on the hepacivirus HCV, a luciferase-encoding reporter virus was used, and virus-mediated intracellular luciferase expression was measured as a marker for viral replication. Furthermore, to rule out the possibility that K22 has an impact on the luciferase reporter activity, a nonreporter HCV construct (HCV Jc1) was employed and quantitative real-time PCR (qRT-PCR) analysis of HCV RNA levels was performed (Fig. S2). In addition, the viral supernatant from infected, K22-treated cells was harvested and used to inoculate naive target cells before the latter were lysed and luciferase reporter activity was determined (infectivity). Similar to the findings for WNV and USUV, K22 concentrations of ≥20 μM led to a significant reduction of viral replication as well as infectivity (Fig. 3a and b and S2). However, 3-(4,5-dimethylthiazol-2-yl)-2,5-diphenyltetrazolium bromide (MTT) cell proliferation assays indicated that the cells used for infection were affected by K22 concentrations of ≥20 μM (Fig. 3c). Nevertheless, a 50% cytotoxic concentration (CC_50_) value (21.3 μM) 3-fold higher than the IC_50_ (7.0 μM) (Table 2) revealed an antiviral effect of K22 on HCV which could be distinguished from the effect on cell viability. Nevertheless, careful interpretation of the data is still required, as the antiviral effect displayed by K22 may be overestimated in the context of HCV infections. Two biotypes of BVDV strain Suwa were tested for their susceptibility to K22 treatment. Viral RNA levels were determined by qRT-PCR as a correlate for viral replication, and viral titers in the supernatant were assessed by titration. Similar to the findings for HCV, K22 concentrations of ≥20 μM and ≥40 μM were necessary to significantly reduce the viral replication of Cp BVDV strain Suwa (BVDV SuwaCp) (Fig. 3e) and Ncp BVDV strain Suwa (BVDV SuwaNcp) (Fig. 3g), respectively. Viral titers were significantly reduced upon treatment with 20 to 50 μM K22 for BVDV SuwaCp (Fig. 3f), with the IC_50_ being 14.2 μM (Table 2), whereas at least 40 μM K22 was necessary to significantly reduce the infectivity of BVDV SuwaNcp, with the IC_50_ being 21.4 μM (Fig. 3h; Table 2). Of note, even though no cellular toxicity resulting from K22 incubations at the concentrations indicated above could be measured, cell proliferation was affected at K22 concentrations of ≥30 μM, as described before (Fig. 3d; Table 2). Collectively, our data show that K22 inhibits a broad range of Flaviviridae members, including JEV, YFV, WNV, USUV, WESSV, and, to a limited extent, HCV and BVDV.

**TABLE 2:** IC_50_ values, CC_50_ values, and selectivity indexes for the effect of K22 treatment on hepacivirus and pestivirus replication and infectivity

Virus	IC_50_ (μM) for:		SI^*a*^	CC_50_ (μM) for:	
	Replication	Infectivity		Proliferation	Cytotoxicity
HCV	7.0	2.2	9.7	21.3	
BVDV SuwaCp	15.5	14.2	>24.6	>350	>1,000
BVDV SuwaNcp	34.7	21.4	>16.4	>350	>1,000

a SI, selectivity index.

### Combination treatment regimens enhance the antiviral activity of K22.

**FIG 4:**
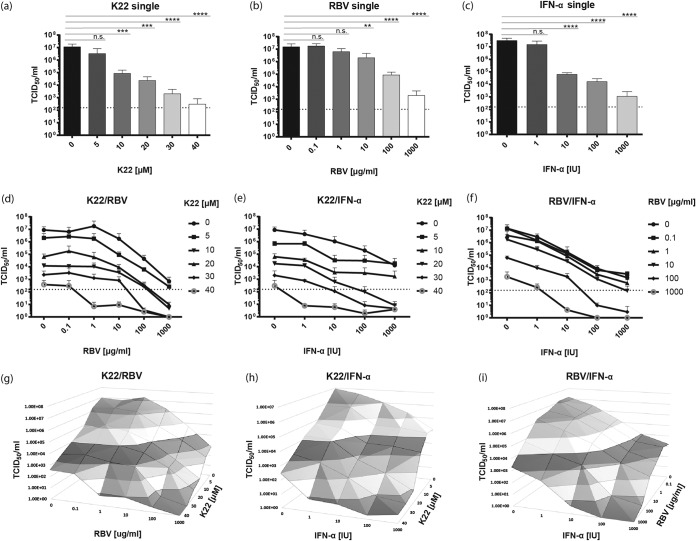
Combination treatment regimens enhance the antiviral effect of K22 on ZIKV. Vero B4 cells were infected with ZIKV (MOI = 0.1 TCID_50_/cell) for 1 h, before the inoculum was discarded and the cells were washed with PBS. The compounds were added at various concentrations. (a) K22 single treatment; (B) RBV single treatment; (c) IFN-α single treatment; (d) K22-RBV combination treatment; (e) K22–IFN-α combination treatment; (f) RBV–IFN-α combination treatment. The cell culture supernatants were collected at 24 h p.i., and viral titers were determined as the number of TCID_50_ per milliliter. The results represent the mean + SD (*n* = 3). The dashed lines indicate the limit of detection. *P* values were determined by 1-way ANOVA followed by Dunnett's multiple-comparison test. ****, *P* ≤ 0.0001; ***, *P* ≤ 0.001; **, *P* ≤ 0.01; n.s., not significant. (g to i) Three-dimensional blots depicting the viral titers for each combination.

Treatment options against members of the family Flaviviridae remain limited, despite the recent success regarding the treatment of HCV infections with the development of direct-acting antivirals (DAA) (14). The recent ZIKV epidemic further underscored the importance of effective viral inhibitors, which can be applicable to rapidly control outbreaks of (re)emerging viruses. Ribavirin (RBV) and interferon alpha (IFN-α) have been employed for their well-documented antiviral activity against several RNA viruses (15). We therefore evaluated the antiviral potential of a combination regimen of K22 together with RBV or IFN-α against ZIKV. To this end, cells were treated either with K22, RBV, or IFN-α alone or with a two drug-combination and subsequently infected for 24 h before the viral titers in the supernatant were determined by a TCID_50_ assay. A single treatment with 5 or 10 μM K22 was sufficient to significantly reduce the viral titers (Fig. 4a). A single treatment with 10 μg/ml RBV moderately reduced the viral titers, whereas increased concentrations of 100 μg/ml and 1,000 μg/ml correlated with significantly reduced viral titers in the supernatant (Fig. 4b). However, high RBV concentrations (100 μg/ml to 1,000 μg/ml) also impacted cell viability (Fig. S3b). IFN-α was effective against ZIKV at doses of ≥10 IU (Fig. 4c), with no effect on cell viability (Fig. S3c). A combination treatment of K22 with either 0.1 or 1 μg/ml RBV did not improve the antiviral effect of K22; however, the addition of at least 10 μg/ml RBV in combination with low K22 concentrations (5 to 10 μM) increased the overall antiviral effect (Fig. 4d and g). Furthermore, a combination of 10 or 20 μM K22 together with 100 μg/ml RBV reduced the viral titers by 4 to 5 orders of magnitude compared to those for the untreated control (Fig. 4d and g). Similarly, combination treatment with doses of ≥10 IU IFN-α together with K22 potentiated the antiviral effect (Fig. 4e and g). In particular, 5 to 10 μM K22 combined with ≥10 IU IFN-α resulted in viral titers reduced by at least 3 orders of magnitude without any further impact on cell proliferation (Fig. 4e and S3e). Combination treatments with RBV and IFN-α at doses of ≥100 IU IFN-α and ≥100 μg/ml RBV increased the antiviral effectiveness against ZIKV compared to that of either single treatment (Fig. 4f). Analysis of the drug combination K22 and RBV at concentrations in the range of the concentrations of each drug that caused only partial inhibition of replication pointed to an additive antiviral effect of the two compounds, indicated by combination indexes (CI) of about 1 (Fig. S4). For K22 combined with IFN-α, a slight synergism was observed for 10 IU IFN-α (⩠6.73 × 10^−6^ μM) and K22 concentrations between 10 and 30 μM, indicated by a CI of <1. In conclusion, K22 treatments in combination with RBV and IFN-α allow the use of reduced K22 concentrations for a level of ZIKV replication impairment similar to that achieved with higher concentrations of K22, RBV, or IFN-α alone while protecting the cells from potential K22- or RBV-induced cytotoxicity when used at high concentrations.

### K22 interferes with membrane reorganization during ZIKV infection.

**FIG 5:**
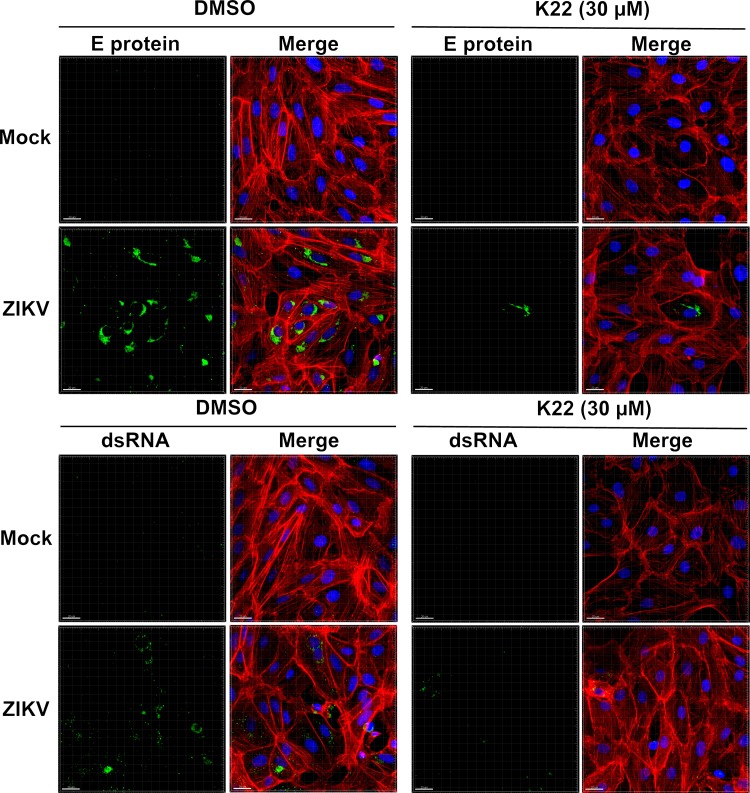
K22 inhibits E protein and dsRNA accumulation in ZIKV-infected cells. Vero cells were pretreated with 30 μM K22 or DMSO for 4 h before infection with ZIKV (MOI = 0.1 TCID_50_/cell). DMSO-treated and noninfected cells (Mock) were included as controls. The inoculum was removed after 1 h, and the cells were washed with PBS. At 48 h p.i., the cells were fixed and processed for immunofluorescence analysis using antibodies directed against flavivirus E protein and dsRNA as markers of ZIKV replication. Cells were counterstained with phalloidin (actin) or DAPI (nuclei). z-projections of multiple optical sections acquired with a Nikon confocal A1 microscope combined with an Eclipse Ti inverted microscope are shown. Bars, 20 μm.

**FIG 6:**
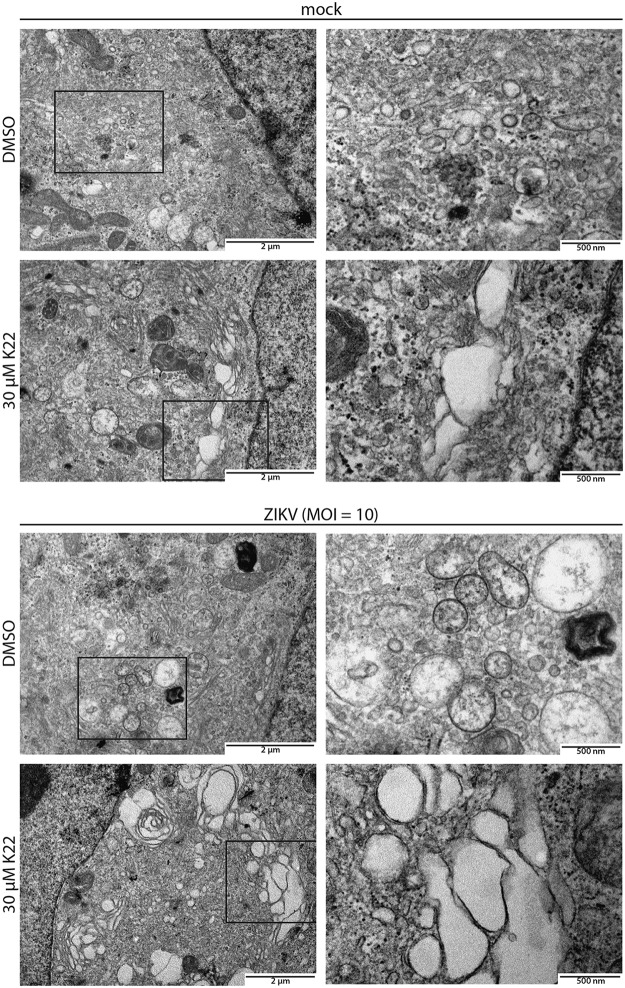
Ultrastructural analysis of the K22-mediated alteration of ZIKV replication compartment formation. Vero B4 cells were pretreated with 30 μM K22 for 2 h and infected with ZIKV (MOI = 10 TCID_50_/cell) for 24 h. The inoculum was removed at 1 h p.i., and the cells were washed with PBS. DMSO-treated and noninfected cells (mock) were included as controls. The cells were fixed at 24 h p.i. and processed for ultrastructural analyses by TEM of ultrathin sections 90 nm thick. Magnifications of representative areas indicated by squares in the left panels are shown on the right.

We have previously reported that K22 prevents the formation of typical HCoV-229E-induced perinuclear DMV clusters, suggesting that K22 interferes with coronavirus replication by impairing membrane-bound replication compartment biogenesis (12). We therefore assessed whether the antiviral effect of K22 observed against Flaviviridae, specifically, against ZIKV, correlates with the impaired establishment of ZIKV-induced replication organelles. To this end, ZIKV-infected cells were fixed at 48 h p.i. and assessed by fluorescence microscopy. Immunofluorescence analysis revealed that the vast majority of cells were infected, as both viral E protein and double-stranded RNA (dsRNA) were efficiently detected in most cells (Fig. 5, left). In concordance with previous results (Fig. 1), both markers of viral infection were severely reduced upon K22 treatment (30 μM), thereby confirming the potent antiviral effect of K22 on ZIKV infections (Fig. 5, right). Additional immunofluorescence analyses with selected flaviviruses (JEV and WNV) further confirmed the antiviral activity of the compound (Fig. S5). Furthermore, ultrastructural ZIKV-induced membrane rearrangements were assessed by transmission electron microscopy (TEM). Despite a vast majority of dimethyl sulfoxide (DMSO)-treated cells being infected (Fig. 5), structures reminiscent of well-described ZIKV membranous replication organelles were only occasionally observed in ZIKV-infected cells (Fig. 6). Fixation methods and cell type-specific determinants likely account for the morphological differences from previously reported ZIKV-induced membranous structures (16). Accordingly, it was also reported that certain replication-associated structures were absent in ZIKV-infected human neural progenitor cells and that vesicular structures were markedly smaller than the ones observed in ZIKV-infected Huh-7 cells (16). In sharp contrast, we repeatedly observed a striking accumulation of large vesicles in the cytosol of ZIKV-infected cells treated with K22, while morphological changes of cellular compartments (dilated vesicular structures) were only occasionally seen in noninfected cells treated with K22 (Fig. 6). These vesicles were notably larger than previously described ZIKV replication organelles (16), as vesicles of at least 500 nm in diameter were frequently observed, and these are unlikely to represent conventional ZIKV-induced replication organelles. Rather, they are suggestive of aberrant structures that do not support ZIKV replication as a result of K22 treatment.

Taken together, we observed an alteration of the intracellular membrane organization upon ZIKV infection in combination with K22 treatment associated with a strong reduction of intracellular levels of dsRNA and E protein. Even though the precise mechanism of action of K22 remains to be elucidated, interference with membrane remodeling functions and membrane-bound RNA replication provides a plausible explanation for the observed pan-antiviral effects.

## DISCUSSION

Many members of the family Flaviviridae are important human and veterinary pathogens. Especially, several members of the Flavivirus genus are considered (re)emerging viruses, which are defined as pathogens that have existed previously or have newly appeared in a population and are increasing in incidence or geographic range (17). Successful treatment of HCV infections has been achieved in the last couple of years with the development of highly effective direct-acting antiviral (DAA) therapies (18). Moreover, favipiravir, also known as T-705 or Avigan, is in use against YFV and WNV infections (as well as infections caused by arenaviruses, bunyaviruses, and alphaviruses) (19), and host-directed compounds, such as cyclophilin inhibitors (20, 21) and α-glucosidase inhibitors (22), have been evaluated in clinical trials to control HCV and dengue virus (DENV) infections, respectively. Furthermore, prophylactic options, such as vaccination, have been developed for YFV, JEV, and tick-borne encephalitis virus (TBEV) (23), as well as for DENV, although the vaccine against DENV suffers from limited efficacy (24, 25). On the other hand, vaccines for humans to combat HCV, WNV, and ZIKV are not available (26). Taken together, the available panel of prophylactic and antiviral treatment options against flavivirus infections in human and animals remains limited, thus emphasizing the need for the development of effective and reliable drugs/vaccines.

Following a screening which aimed at identifying anti-HCoV-229E compounds, we have described a potent small-compound inhibitor, K22, which efficiently inhibited coronavirus replication and was recently shown to additionally inhibit a wide range of virus species within the families Coronaviridae and Arteriviridae (12, 13). K22 has been shown to act on early postentry stages of coronavirus replication and to target membrane-bound RNA synthesis by interfering with the establishment of virus-induced replicative structures. Importantly, HCoV-229E mutants that were able to replicate in the presence of K22 were isolated following several virus passages on K22-treated cells. These escape mutants contained amino acid substitutions in nonstructural protein 6 (nsp6; H121L, M159V) that were associated with K22 resistance. Coronavirus nsp6 contains multiple membrane-spanning domains and is essential to membrane diversion events leading to the biogenesis of replication organelles (27). These findings thereby corroborate a critical step inhibited by K22 during membrane remodeling events.

Given these findings, we speculated that K22 might offer potential as a panviral inhibitor, considering that replication strategies are highly similar between members of the family Flaviviridae and other positive-strand RNA viruses. Indeed, we show here that several members of the Flaviviridae are efficiently inhibited by K22, with these viruses including ZIKV, JEV, YFV, WNV, USUV, WESSV, and, to a limited extent, HCV and BVDV. Moreover, combination treatment regimens with the approved broad-acting antivirals RBV and IFN-α supported the antiviral effect of K22 on ZIKV. This finding implies not only that effective K22 concentrations can be substantially reduced but also that the therapeutic window of K22 can be improved. Our results should also encourage efforts to improve the antiviral activity of K22 by assessing further chemical modifications to reduce possible cytotoxicity and the impact of K22 on cell proliferation and thereby enhance the therapeutic range of K22.

Compartmentalization of the viral replication machinery within host-derived endomembrane structures is a conserved hallmark among positive-sense RNA viruses (11, 28, 29) and is hypothesized to provide optimized concentrations of macromolecules and to coordinate the regulated succession of events required for efficient viral replication. Furthermore, compartmentalization protects viral replication intermediates, such as dsRNA, from recognition by cytosolic pattern recognition receptors that elicit the first steps of a host innate immune response (30–32). Time-of-addition experiments in the context of ZIKV revealed that K22 acts at a postentry stage, presumably during the establishment of viral replication in the cytosol of the infected cell, reminiscent of the suggested mode of action in the context of HCoV-229E inhibition (12). Subsequent ultrastructural analyses of ZIKV-infected cells following K22 treatment revealed the predominant appearance of aberrant vacuoles in the cytosol of infected cells associated with the pronounced reduction of perinuclear dsRNA accumulation observed by fluorescence imaging. We therefore speculate that K22 interferes with the biogenesis of replicative organelles by prematurely abrogating membrane diversion functions leading to functional replicative structures. However, whereas K22 completely prevented the formation of coronavirus replicative organelles (12), K22 appeared to increase membrane proliferation and the vesicle size of ZIKV-induced replication structures. Whether this suggests either different mechanisms by which K22 interferes with the formation of functional replication complexes or the action of the same mechanism with a different outcome remains to be elucidated.

In conclusion, we describe the antiviral activity of a small-compound inhibitor, K22, against several members of the family Flaviviridae, in addition to the previously reported antiviral effect against members of the family Coronaviridae and other nidoviruses. K22 therefore represents a potential panviral inhibitor and could complement current intervention strategies against several (re)emerging viral diseases.

## MATERIALS AND METHODS

### Cell culture and viral strains.

The present work included several flaviviruses: a low-passage-number JEV Laos strain belonging to genotype 1 (G1; CNS769/Laos/2009; GenBank accession number KC196115.1; kindly provided by R. Charrel, Aix-Marseille Université, Marseille, France), a low-passage-number ZIKV clinical isolate (<5 passages) of the Asian lineage obtained from a viremic patient in Puerto Rico in 2015 (PR-2015; PRVABC59; GenBank accession number KX377337; obtained from Public Health England [PHE]), WNV NY99 (GenBank accession number DQ211652.1) and the high-passage-number African lineage USUV SAAR-1776 strain (GenBank accession number AY453412) (both WNV NY99 and USUV SAAR-1776 were kindly provided by R. Hoop, Institute of Veterinary Bacteriology University of Zürich, Zürich, Switzerland), WESSV strain SAH-177 99171-2 (GenBank accession number EU707555.1; obtained from European Virus Archive goes global [EVAg]), and finally, the YFV 17D strain used in the Stamaril vaccine (GenBank accession number X03700). The JEV, WNV, USUV, and YFV strains were propagated in Vero cells (ATCC CCL81; American Type Culture Collection, VA, USA) in Glasgow minimum essential medium (G-MEM-BHK-21; Gibco, Thermo Fisher Scientific, Reinach, Switzerland) supplemented with 2% (vol/vol) fetal bovine serum (FBS; Biowest, Nuaillé, France) and cultured at 37°C in a 5% CO_2_ atmosphere. ZIKV and WESSV were was passaged in Aedes albopictus C6/36 cells cultured in G-MEM-BHK-21 (Gibco) supplemented with 2% (vol/vol) FBS (Biochrom; Bioswisstec AG, Schaffhausen, Switzerland) at 28°C in 5% CO_2_. HCV JcR-2a stocks were obtained by electroporation of Huh-7.5 cells with *in vitro*-transcribed HCV RNA, as previously described (33). JcR-2a is a full-length recombinant HCV genome based on the Jc1 intragenotypic HCV chimera (34) and, further, encodes the Renilla luciferase reporter gene (35). Huh7-Lunet-N-hCD81-FLuc cell lines were grown at 37°C with 5% CO_2_ in Dulbecco's modified Eagle's medium (DMEM; Invitrogen, Thermo Fisher Scientific) supplemented with 2 mM l-glutamine, nonessential amino acids (NEA), 100 U/ml of penicillin, 100 μg/ml of streptomycin, and 10% (vol/vol) FBS. For the Huh7-Lunet-N-hCD81-FLuc cell line, blasticidin was added to the culture medium at a concentration of 5 μg/ml. BVDV strains SuwaCp and SuwaNcp were isolated at the Institute of Virology and Immunology (36) and passaged on embryonic bovine turbinate (BT) cells as described previously (37) in Eagle minimal essential medium (Gibco) supplemented with 7% (vol/vol) FBS (PAA; PAA Laboratories, GE Healthcare, Glattbrugg, Switzerland), 1% (vol/vol) GlutaMAX (Gibco), 1% (vol/vol) penicillin-streptomycin (Biochrom), 0.5% (vol/vol) neomycin-bacitracin (Biochrom), and 1% (vol/vol) NEA (Biochrom).

### Reagents.

K22 (structural name, (*Z*)-*N*-{3-[4-(4-bromophenyl)-4-hydroxypiperidin-1-yl]-3-oxo-1-phenylprop-1-en-2-yl} benzamide) was purchased from ChemDiv (catalog number 4295-0370; San Diego, CA) and DMSO. RBV was purchased from Sigma-Aldrich (catalog number R9644; Buchs, Switzerland), and recombinant human IFN-αA/D (IFN-α) was purchased from Sigma-Aldrich (catalog number I4401). Stock solutions were prepared in H_2_O and stored according to the manufacturer's recommendations.

### Infection experiments.

To test the effect of K22 on flavivirus, hepacivirus, and pestivirus infection, 8 × 10^4^ Vero cells were seeded in 12-well dishes (flaviviruses), 5 × 10^4^ Huh7-Lunet-N-hCD81-FLuc cells were seeded in 12-well dishes (hepacivirus), or 1 × 10^4^ BT cells were seeded in 96-well dishes (pestivirus). At 1 day postseeding, cells were treated with different concentrations of K22 (diluted in DMSO at the concentrations indicated below) for 3 to 4 h and preincubated in 5% CO_2_ at 37°C. Cells were infected with different viruses at a multiplicity of infection (MOI) of 0.1 TCID_50_/cell for 1 h at 37°C. For HCV infection, cells were infected with JcR-2a or HCV Jc1 for 3 h at 37°C. Afterwards, cells were washed with prewarmed phosphate-buffered saline (PBS) solution, before cell culture medium supplemented with K22 or the DMSO control at each concentration was added, and incubated at 37°C in a 5% CO_2_ atmosphere. At 24 h and/or 48 h postinfection (p.i.), supernatants and cells were collected for further analysis.

### Flavivirus replication and infectivity.

Flavivirus replication and the cytopathic effect (CPE) were evaluated from collected cells. Cells in suspension were first labeled with the Fixable Aqua dead cell stain kit (Thermo Fisher Scientific, Waltham, MA, USA) for 30 min on ice, followed by washing with cold PBS. Then, the suspended cells were fixed with 4% (wt/vol) paraformaldehyde (PFA) for 10 min on ice and thereafter washed and permeabilized with 0.3% (wt/vol) saponin in PBS supplemented with anti-flavivirus group antigen antibody 4G2 (catalog number HB-112; ATCC) for 15 min on ice. After washing, anti-mouse IgG2a conjugated with Alexa Fluor 647 (Thermo Fisher Scientific) was added for 15 min, and the cells were acquired on a FACSCanto II flow cytometer (BD Biosciences, Allschwil, Switzerland). FlowJo (v.9.1) software (TreeStar, Inc., Ashland, OR, USA) was used for analysis. Cell debris and doublets were excluded by electronic gating in forward/side scatter plots. Cell culture supernatants were collected at 24 and 48 h p.i., and the flavivirus titers in Vero cells were determined using the immunoperoxidase monolayer assay (IPMA). For that, supernatants 10-fold diluted in cell culture medium supplemented with 2% (vol/vol) FBS were incubated on Vero cells for 72 h at 37°C in a 5% CO_2_ atmosphere. At that point, cells were fixed in 4% (wt/vol) PFA for 10 min at room temperature, washed, and immunolabeled with anti-flavivirus group antigen antibody 4G2 diluted in 0.3% (wt/vol) saponin in PBS for 30 min at 37°C and then washed and incubated for another 30 min at 37°C with goat anti-mouse IgG conjugated with horseradish peroxidase (Dako), followed by incubation for 10 to 30 min at room temperature with 3-amino-9-ethylcarbazole (AEC) substrate (Sigma-Aldrich). Titers were calculated and expressed as the number of TCID_50_ per milliliter using the Reed and Muench method (38).

### Hepacivirus replication and infectivity.

Cells were lysed at 48 h p.i. in 150 μl passive lysis buffer (Promega) per well for the assessment of the Renilla luciferase (viral replication) activities. The details of the Renilla luciferase assay have been described elsewhere (39). For the determination of HCV Jc1 replication, infections were performed as described above, but with Jc1 virus and an MOI of 1 or 0.1 TCID_50_/cell. Cells were lysed at 24 h p.i. for RNA extraction using a NucleoSpin RNA kit (Macherey-Nagel, Düren, Germany) according to the manufacturer's instructions. qRT-PCR of HCV RNA was performed in duplex with a GAPDH (glyceraldehyde-3-phosphate dehydrogenase) calibrator in a LightCycler 480 instrument (Roche, Mannheim, Germany) as described previously (40) but with 45 cycles and the following primers and probes: primer HCV S-147 (5′-TCTGCGGAACCGGTGAGTA-3′), primer HCV A-221 (5′-GGGCATAGAGTGGGTTTATCCA-3′), an HCV probe (5′-6FAM-AAAGGACCCAGTCTTCCCGGCAA-TMR [where 6FAM is 6-carboxyfluorescein and TMR is 6-carboxytetramethylrhodamine]), primer GAPDH S (5′-GAAGGTGAAGGTCGGAGTC-3′), primer GAPDH A (5′-GAAGATGGTGATGGGATTTC-3′), and a GAPDH probe (5′-LC640-CAAGCTTCCCGTTCTCAGCCT-BHQ-1) (where LC40 is LightCycler 640 [Roche Diagnostics] and BHQ-2 is black hole quencher 2). The amount of viral RNA relative to that in the control sample (DMSO-treated cells) was calculated using the ΔΔ*C_T_* threshold cycle (*C_T_*) method with GAPDH as an internal control (41). Supernatants were used to inoculate naive Huh7-Lunet-N-hCD81-FLuc cells that had been seeded the day before at 5 × 10^4^ cells per well in 12-well dishes. The cells were lysed at 72 h p.i. in 150 μl passive lysis buffer (Promega) per well for the assessment of the Renilla luciferase activity (infectivity).

### Pestivirus replication and infectivity.

Cells were lysed at 48 h p.i., and intracellular RNA was extracted using a NucleoMag 96 RNA kit (Macherey-Nagel, Oensingen, Switzerland) on a Kingfisher Flex system according to the manufacturer's recommendation. qRT-PCR was performed as described previously (42) using a QuantiTect probe RT-PCR kit (Qiagen, Hombrechtikon, Switzerland) according to the manufacturer's recommendations, and amplification was detected using a TaqMan 7500 real-time PCR system (Applied Biosystems, Thermo Fisher Scientific). The amount of viral RNA relative to that in the control sample (DMSO-treated cells) was calculated using the ΔΔ*C_T_* method with GAPDH as the internal control (41). Viral titers were determined by serial dilution on BT cells and incubation at 37°C in 5% CO_2_ for 4 days. BVDV SuwaCp titers were determined by microscopy upon quantification of the cytopathic effect (CPE). BVDV SuwaNcp titers were determined upon fixation, followed by immunoperoxidase (IPO) staining as described previously (37) using an AEC staining kit (catalog number AEC101-1KT; Sigma-Aldrich), and viral titers were calculated and expressed as the number of TCID_50_ per milliliter using the Spearman-Kärber method.

### Cell toxicity and proliferation assays.

To evaluate the effect of K22 on cell viability, proliferation, as well as cytotoxicity, uninfected cells were treated with the compound at the concentrations specified above or with DMSO as a control. To determine the effect of K22 on cell viability, Vero cells were collected and labeled with the Fixable Aqua dead cell stain kit (Thermo Fisher Scientific) for 30 min on ice, before the cells were washed with cold PBS and fixed for flow cytometry analysis. As a positive control for cell death, cells were heated at 65°C for 30 min. The mean fluorescence intensity (MFI) was determined by flow cytometry analysis on a FACSCanto II flow cytometer (BD Bioscience) and evaluated using FlowJo (v.9.1) software. To determine the cytotoxicity of K22, the CytoTox 96 nonradioactive cytotoxicity assay (catalog number G1780; Promega) was used according to the manufacturer's recommendations. To this end, 1 × 10^4^ Vero B4 cells (kindly provided by M. Müller, University of Bonn Medical Centre, Bonn, Germany) or BT cells were seeded in a 96-well format. The compound was added at the concentrations indicated above, and cytotoxicity was determined at 24 and/or 48 h p.i. by determination of the absorbance at 490 nm using a plate reader (VersaMax [Molecular Devices, Wokingham, UK] or an EnSpire 2300 multilabel reader [PerkinElmer, Schwerzenbach, Switzerland]). Cell proliferation following compound treatment was determined using a MultiTox-Fluor multiplex cytotoxicity assay (catalog number G9200; Promega) according to the manufacturer's recommendations. The readout was performed using a multilabel reader (EnSpire 2300 multilabel reader; PerkinElmer) upon excitation at ∼400 nm and emission at ∼505 nm. To measure the effects on Huh7-Lunet-N-hCD81-FLuc cell viability, an 3-(4,5-dimethylthiazol-2-yl)-2,5-diphenyltetrazolium bromide (MTT) cell proliferation assay was performed at 48 h posttreatment. Briefly, an MTT stock solution was prepared by dissolving the compound (catalog number M5655; Sigma) at 5 mg/ml in PBS, and the stock was diluted 10 times in prewarmed complete DMEM. The cell supernatant was replaced by the diluted MTT solution (50 μl/well), and the cells were incubated for 1 h at 37°C. The cell supernatant was removed and replaced by 50 μl/well DMSO to dissolve the precipitate, and the absorbance was read at 570 nm.

### Time-of-addition assay.

To study the effect of K22 on early virus-cell interaction, 8 × 10^4^ Vero B4 cells were seeded in 12-well plates in DMEM (Gibco) supplemented with 10% (vol/vol) FBS, 1% (vol/vol) NEA (Gibco), and 1% (vol/vol) penicillin-streptomycin (Invitrogen) and allowed to reach 80% confluence overnight. Subsequently, K22 at various concentrations was added at specific time points relative to the time of infection with ZIKV, and the cell culture supernatants were collected at 24 h p.i. for measurement of virus release. Shortly, the compound was diluted in DMSO to reach the desired concentrations (0, 5, 10, 20, 30, and 40 μM) and incubated with the cells for 2 h prior to infection, during infection, or after a period of 2, 6, 10, 14, 18, 22, or 24 h. The infection with ZIKV was conducted as follows: cell culture medium was removed and replaced by 1 ml of DMEM containing ZIKV diluted to an MOI of 0.1 TCID_50_/cell, and the cells were incubated for 1 h at 37°C in 5% CO_2_. Next, the inoculum was discarded, the cells were washed with prewarmed PBS, and culture medium was added to the wells. Additionally, in order to analyze the direct effect of K22 on virus stability, ZIKV in DMEM was incubated for 2 h at room temperature with the different K22 concentrations and titrated in 10-fold serial dilution on Vero cells for measurement of virus infectivity as described above.

### Combination treatment.

To evaluate the effects of the combination treatment regimens on ZIKV infectivity, 4 × 10^4^ Vero B4 cells were seeded in 24-well plates in DMEM (Gibco) supplemented with 10% (vol/vol) FBS, 1% (vol/vol) NEA (Gibco), and 1% (vol/vol) penicillin-streptomycin (Invitrogen) and allowed to reach 80% confluence overnight. The cells were infected with ZIKV at an MOI of 0.1 TCID_50_/cell at 37°C in 5% CO_2_ for 1 h, before the inoculum was discarded and the cells were washed with prewarmed PBS. Compounds were added at various concentrations (for K22 diluted in DMSO, 0, 5, 10, 20, 30, or 40 μM; for RBV diluted in H_2_O, 0.1, 1, 10, 100, and 1,000 μg/ml; for IFN-α diluted in H_2_O, 1, 10, 100, or 1,000 IU/500 μl) either as single agents or in combination. The cell culture supernatants were collected at 24 h p.i., and the viral titers were determined using the IPMA as described above and calculated as the number of TCID_50_ per milliliter. Cell proliferation upon combination treatment was determined using the MultiTox-Fluor multiplex cytotoxicity assay (Promega) as described above.

### Immunofluorescence analysis.

Vero cells were seeded on 8-chamber Lab-Tek II slides (Nunc; Milian, Geneva, Switzerland) and pretreated with 30 μM K22 or DMSO for 4 h. Cells were infected with ZIKV, JEV, and WNV at an MOI of 0.1 TCID_50_/cell in medium supplemented with 30 μM K22 or equivalent volumes of DMSO as a control. Cells were washed with PBS at 1 h p.i. and incubated in medium supplemented with 30 μM K22 or equivalent volumes of DMSO for an additional 48 h. Cells were fixed with 4% (wt/vol) PFA, permeabilized, and blocked with a PBS solution supplemented with 1% (vol/vol) FBS and 0.3% (wt/vol) saponin. E protein and dsRNA replication intermediates were detected using anti-flavivirus group antigen antibody (1:10; catalog number HB-112; ATCC) and anti-dsRNA J2 MAb (1:200; English and Scientific Consulting). Alexa Fluor 488-coupled anti-mouse IgG2a antibodies were used as secondary antibodies. Alexa Fluor 633-coupled phalloidin (Invitrogen) and DAPI (4′,6-diamidino-2-phenylindole) were used to counterstain the actin cytoskeleton and nuclei, respectively. Slides were mounted in Mowiol mounting medium. For microscopy analysis, a Nikon confocal A1 microscope (Nikon AG, Egg, Switzerland) combined with an Eclipse Ti inverted microscope (Nikon) and digital imaging Nikon software (NIS-Elements AR, v.3.30.02) were used. Image acquisitions were performed in stacks of 0.2 μm over at least a 10-μm thickness using a 40× objective, and sequential and not simultaneous channel acquisition was employed; in order to give high-resolution images, the acquisition setting was performed with an optimized voxel size and an automatic threshold. The images were analyzed with Imaris (v.8.0.2) software (Bitplane AG, Zurich, Switzerland). To avoid false-positive emissions, different settings, including those for background subtraction, threshold applications, gamma correction, and maxima, were applied.

### Transmission electron microscopy.

Vero B4 cells were seeded in 24-well plates and pretreated with 30 μM K22 or DMSO for 2 h. Cells were infected with ZIKV (MOI = 10 TCID_50_/cell) in medium supplemented with 30 μM K22 or equivalent volumes of DMSO. Cells were washed with PBS at 1 h p.i. and incubated in medium supplemented with 30 μM K22 or equivalent volumes of DMSO. Processing of samples for TEM was performed as described by Schätz et al. (43). Shortly, cells were fixed at 24 h p.i. with 2.5% (wt/vol) glutaraldehyde (Merck, Darmstadt, Germany) in 0.1 M cacodylate buffer (Merck, Hohenbrunn, Germany), pH 7.4, and postfixed with 1% (wt/vol) OsO_4_ (Chemie Brunschwig, Basel, Switzerland) in 0.1 M cacodylate buffer. Thereafter, cells were dehydrated in an ascending ethanol series (70%, 80%, 90%, 94%, 100%, 100%, 100% [vol/vol] for 20 min each) and embedded in Epon resin, which is a mixture of Epoxy embedding medium, dodecenyl succinic anhydride (DDSA), and methyl nadic anhydride (MNA) (Sigma-Aldrich, Buchs, Switzerland). Ultrathin sections of 90 nm were then obtained with diamond knives (Diatome, Biel, Switzerland) on a Reichert-Jung Ultracut E microtome (Leica, Heerbrugg, Switzerland) and collected on collodion-coated 200-mesh copper grids (Electron Microscopy Sciences, Hatfield, PA, USA). Sections were double stained with 0.5% (wt/vol) uranyl acetate for 30 min at 40°C (Sigma-Aldrich, Steinheim, Germany) and 3% (wt/vol) lead citrate for 10 min at 20°C (Laurylab, Saint Fons, France) in Ultrastain (Leica, Vienna, Austria) and examined with a Philips CM12 TEM (FEI, Eindhoven, The Netherlands) at an acceleration voltage of 80 kV. Micrographs were captured with a Mega View III camera using iTEM software (v.5.2; Olympus Soft Imaging Solutions GmbH, Münster, Germany).

### Statistical evaluation.

Testing of the statistical significance of the differences in the mean values was performed using GraphPad Prism (v.7.04) software for Windows (GraphPad Software, La Jolla, CA, USA). Either one-way or two-way analysis of variance (ANOVA) was used, followed by Dunnett's multiple-comparison testing. IC_50_s and IC_90_s were calculated from nonlinear regression analyses with the least-squares fit method, and the upper and lower plateaus were set to 100 and 0, respectively. Significant outliers were removed after performing Grubbs' test (QuickCalcs; GraphPad). Drug combination analysis was performed using CompuSyn, a computer program for quantitation of the synergism and antagonism of drug combinations (44).

## Supplementary Material

Supplemental file 1
