# Supplementary material for: The Small-Compound Inhibitor K22 Displays Broad Antiviral Activity against Different Members of the Family Flaviviridae and Offers Potential as a Panviral Inhibitor
Source: Antimicrob Agents Chemother. 2018 Oct 24;62(11):e01206-18. doi: 10.1128/AAC.01206-18 (PMC6201103; doi:10.1128/AAC.01206-18)
Supplement: Supplemental file 1 [file zac011187603s1.pdf]

Figure S1

Cell viability and cytotoxicity after K22 treatment for 48 h. Commercially available cell toxicity assays (cytoxcicity; CytoTox 96® Non-Radioactive Cytotoxicity Assay, Promega) as well as viability assays (proliferation; MultiTox-Fluor Multiplex Cytotoxicity Assay, Promega) have been performed to determine the cytotoxic effect of K22 on Vero B4 cells. Data are normalized to DMSO treated cells. Depicted are the values 48 h post treatment, mean ± SD, n=3.

Figure S2

K22 reduces HCV Jc1 replication. Lunet N hCD81 Fluc cells were pre-treated with K22 for 3 h before supernatant removal and infection with Jc1 virus for 3 h at 37°C at an MOI of 1 or 0.1. After this, the inoculum was removed and the compounds added and left until the end of the experiment. Cells were lysed 24 h post-infection and intracellular RNA extracted for qRT-PCR. Viral replication was calculated using the ΔΔCT method and is depicted as fold induction compared to DMSO control. Mean (bar) + SD, n=3-4. (****, p≤ 0.0001, ***, p≤ 0.001; **, P≤0.01; *, P≤0.05; 1-way ANOVA followed by Dunnett's multiple comparison test, means + SD; n.s. not significant).

Figure S3

Cell viability after combination treatment regimens. Vero B4 cells were treated with compounds at various concentrations. (a) K22 single treatment, (B) RBV single treatment, (c) IFN-alpha single treatment, (d) K22/RBV combination treatment, (e) K22/IFN-alpha combination treatment and (f) RBV/IFN-alpha combination treatment. A commercially available cell viability assay (MultiTox-Fluor Multiplex Cytotoxicity Assay, Promega) has been performed 24 h post treatment to determine the effect of compound treatment on cell

viability. Data are normalized to DMSO treated cells. Depicted are the values 48 h post treatment, mean ± SD, n=3.

Figure S4

Drug Combination Analysis of different combination treatment regimens. Combination indices (CI) in the ranges of only partial nhibition of replication by both drugs were calculated using Drug Combination Analysis implemented in CompuSyn. CI around 1 indicates additive effects of the two drugs applied (K22 and RBV, K22 and IFN respectively). CI > 1 point to antagonistic effects, while CI < 1 suggest synergistic behavior.

Figure S5

K22 inhibits E protein and dsRNA accumulation in flavivirus-infected cells. Vero cells were pretreated with 30 µM K22 or DMSO for 4 h before infection with JEV or WNV (MOI of 0.1 $TCID_{50}$/cell). DMSO-treated and non-infected cells (mock) were included as controls. Inoculum was removed after one hour and cells were washed with PBS. 48 h p.i., cells were fixed and processed for immunofluorescence analysis using antibodies directed against flavivirus E protein and dsRNA as markers of viral replication. Cells were counterstained with phalloidin (actin) or DAPI (nuclei). Z-projections of multiple optical sections acquired with a Nikon confocal A1 combined with an ECLIPSE Ti inverted microscope are shown. Scale bar: 20 µm.

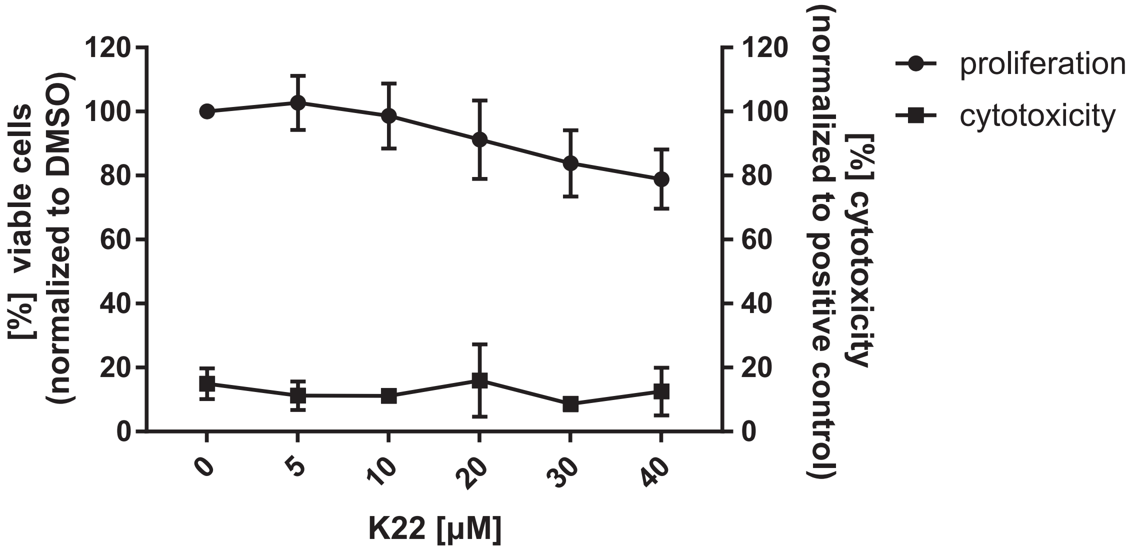

[%] viable cells
(normalized to DMSO)
[%] cytotoxicity
(normalized to positive control)
120
100
80
60
40
20
0
0
5
10
20
30
40
K22 [µM]
proliferation
cytotoxicity

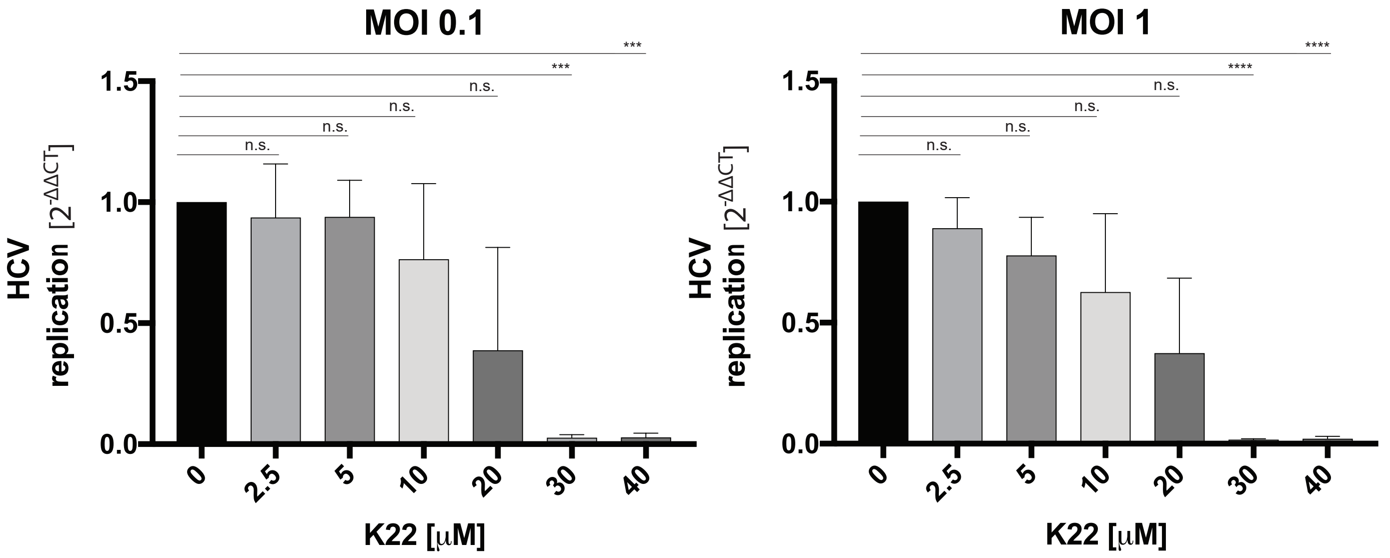
MOI 0.1
MOI 1
HCV replication [2^-ΔΔCT]
K22 [μM]
0
2.5
5
10
20
30
40
0.0
0.5
1.0
1.5
n.s.
***
****

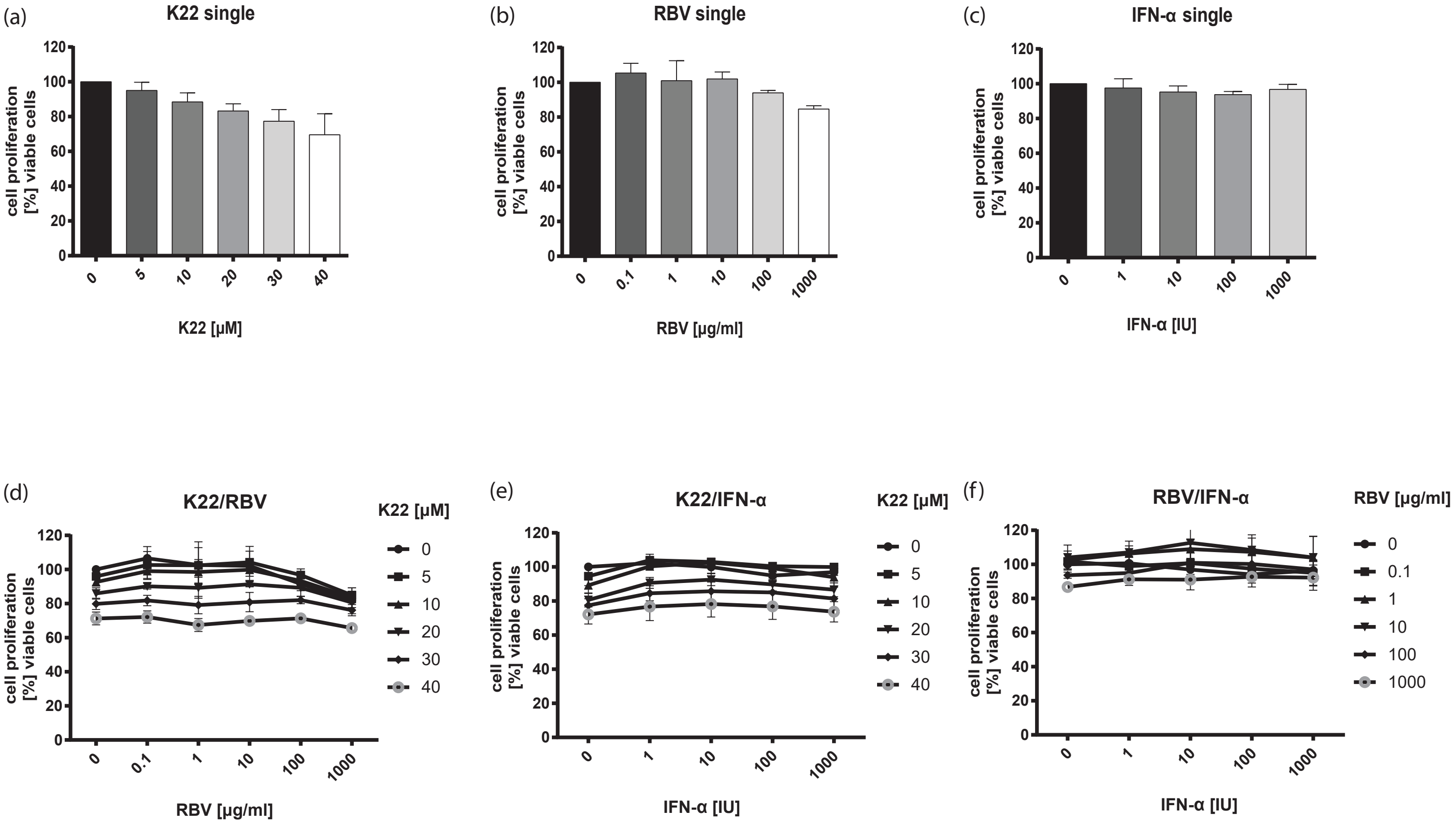
(a)
K22 single
cell proliferation [%] viable cells
120
100
80
60
40
20
0
0
5
10
20
30
40
K22 [µM]
(b)
RBV single
0
0.1
1
10
100
1000
RBV [µg/ml]
(c)
IFN-α single
0
1
10
100
1000
IFN-α [IU]
(d)
K22/RBV
K22 [µM]
0
5
10
20
30
40
RBV [µg/ml]
(e)
K22/IFN-α
K22 [µM]
IFN-α [IU]
(f)
RBV/IFN-α
RBV [µg/ml]
0
0.1
1
10
100
1000
IFN-α [IU]

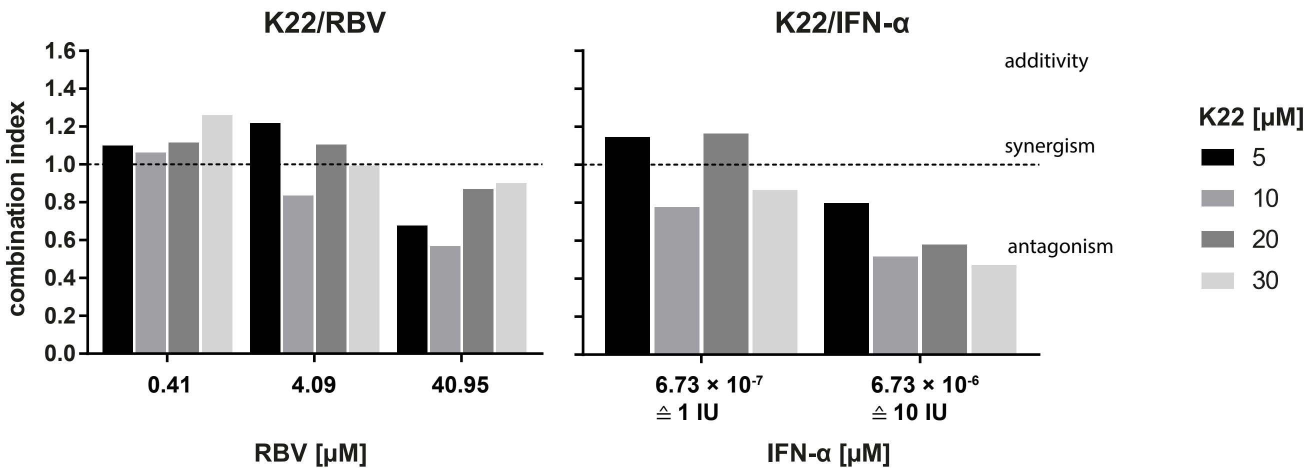
K22/RBV
K22/IFN-α
combination index
1.6
1.4
1.2
1.0
0.8
0.6
0.4
0.2
0.0
0.41
4.09
40.95
RBV [µM]
6.73 × 10-7
≙ 1 IU
6.73 × 10-6
≙ 10 IU
IFN-α [µM]
additivity
synergism
antagonism
K22 [µM]
5
10
20
30

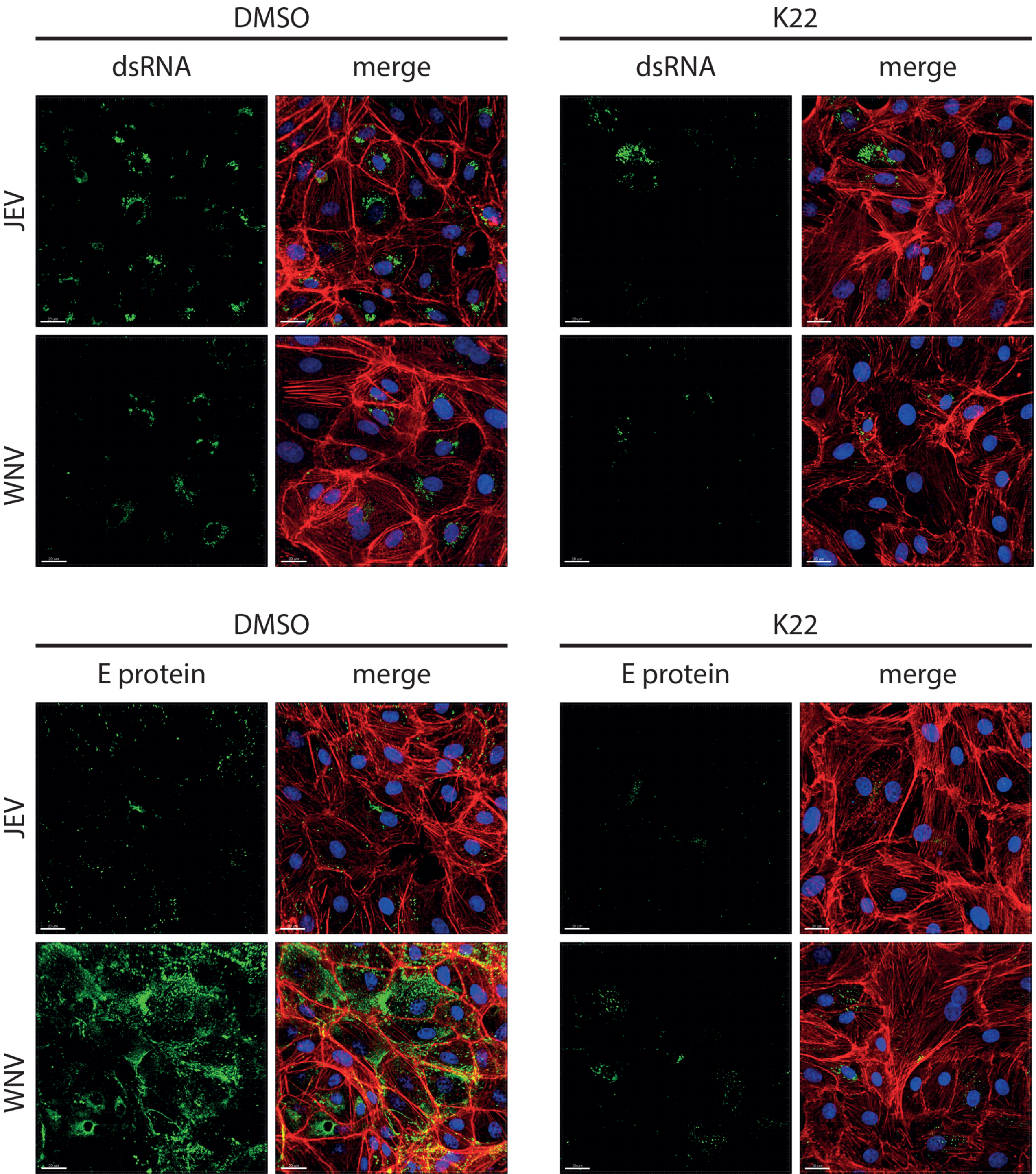
DMSO
K22
dsRNA
merge
dsRNA
merge
JEV
WNV
DMSO
K22
E protein
merge
E protein
merge
JEV
WNV
